# Clinic of the Jefferson Medical College

**Published:** 1852-05

**Authors:** J. Aitken Meigs


### CLINICAL REPORTS.

*Clinic of the Jefferson Medical College. Service of* PROFESSOR DUNGLISON.

(Reported by J. AITKEN MEIGS, M. D.)

*January* 24th, 1852.

The attention of the class was directed to the consideration of a highly important set of affections.

Cardiac disease is at all times the subject of painful interest to the persons affected, and not unfrequently the cause of considerable perplexity to the practitioner. Indeed, the least disturbance of the normal action of the heart is very often amply sufficient to fill the mind of the patient with intense alarm and anxiety. He at once supposes that he is the victim of a confirmed and incurable organic disease; whereas, when properly investigated, the symptoms are most frequently found to be evidences of some temporary excitement of the central organ of the circulation, produced by an unusual excitation or depression of the system generally. Of the great number of persons presenting themselves for medical examination and advice at the clinic of this institution, and complaining of some derangement of the heart's action, either as to frequency, force, or rhythm, comparatively few offer the evidences of a confirmed and well-marked structural disease of that organ. The majority of such cases are mainly dependent upon faulty innervation; being traceable to functional aberration on the part of some particular organ or set of organs, or to a plethoric, anæmic or depraved condition of the circulatory fluid.

Nevertheless, organic affections of the heart, since they have become the objects of careful research, and elaborate study, are found to be much more common than was formerly supposed; and you will recollect, that during the present clinical course, we have, in your presence, examined and prescribed for several cases, illustrative of various morbid conditions of the muscular tissue, and certain structural alterations of the valvular apparatus of the heart.

You see, then, the importance of a thorough study of these diseases, the necessity of mastering them in all their details, and obtaining some clear and definite conceptions as to the differential diagnosis between purely functional and essentially structural diseases of the heart; since mistakes in this respect will often be productive of much needless, and occasionally serious alarm, in the minds of the parties interested. Moreover, the success of our therapeutical applications, and the favorable or unfavorable character of the prognosis, will depend intimately upon the correctness and exactitude of our diagnosis.

I propose here to introduce for our mutual study and instruction, the following interesting series of cases, elucidative of a few of the prominent features of both functional and organic diseases of the heart; and illustrating not only the variety of causes which may give rise to these morbid conditions, but also the difficulty of diagnosis, and the consequent difficulty of successful and rational treatment—too often, indeed, unsuccessful even when the characters of the diseased condition have been accurately appreciated.

Several of these cases are examples of cardiac palpitation, some of which are purely functional in character, appearing to be unassociated with any other disease, while the others are more or less complicated. Of the complicated cases there is one of bronchitis and perhaps of tuberculous deposition; another, in some manner connected with the rheumatic diathesis; and a third, dependent upon torpor of the colon. Three of our patients are affected with organic disease of the heart. In one there is mitral insufficiency; in another, dilatation of the organ; in another, hypertrophy with auriculo-ventricular derangement; and, in a fourth, hypertrophy with disease of the semilunar valves.

Having detailed these cases *seriatim*, we shall consider the differential diagnosis between these two classes of heart affection.

Case I. Mary D——, æt. 25, of a pale complexion, light hair, middling stature, and unmarried, in here presenting herself for relief, complains, as you have heard, of intense pain in the præcordial region, radiating from the heart as a centre over the entire chest. She has been troubled with this pain, at varying intervals, for the last two years. No unusual motion of the heart was at first experienced, but a year ago last Christmas she began to be aware of slight palpitation accompanying the pain, and paroxysmal in character. Latterly, the paroxysms of palpitation have increased so much in frequency and violence, that not only is her mind filled with great anxiety as to the result of her malady, but she suffers considerable physical distress, as you perceive, from the consequent dyspnœa.

The patient attributes her present ill health to the grief and trouble which she endured, in consequence of the loss of a sister to whom she was much attached.

The attack at first came on whenever she made any unusual exertion, or was more than ordinarily excited, depressed, or in any way agitated in mind. At the present time, the least exercise, as in the performance of her customary domestic avocations, is productive of a paroxysm of severe palpitation and its attendant dyspnœa. The oppression experienced at the præcordium during these attacks is occasionally so great, especially when she lies down, as to endanger suffocation.

She is also, at times, seized with vomiting or regurgitation without any preceding sickness or uncomfortable sensation; and is furthermore attacked, at irregular periods, with severe pain in the middle of the back. Her appetite is diminished; tongue pale, exanguious in appearance, and quivering when projected; bowels slightly constipated, more especially during the past few weeks; and this condition is, now and then, associated with acid or bitter eructations, and frequent discharges of flatulency.

Thus much for the general survey of the case. Let us seek further information from the aids furnished by the ear and the hand.

Percussion elicits no unusual or extended dulness, from which we legitimately infer that there is no enlargement of the heart; and, therefore, that the aberration in the action of the organ is not properly assignable to, or in any manner connected with, hypertrophy or augmentation of size in the organ eccentrically. Auscultation affords confirmation to the inference, that hypertrophy does not exist; for we find that the cardial pulsations, though evidently increased in frequency, are diminished in force, the systole of the heart being characterized by an irregular and fluttering effort; phenomena the reverse of those presented by thickness of the parietes of the organ. Furthermore, there is a diminution of the sounds, and a general tremulous diffusion of the impulse, which is observed over a wider space, though certainly much weaker and less decided than in health.

It may be well to observe in this connection, that in the majority of cases of cardial palpitation, there is an increase instead of a diminution of the sounds, which are at times so loud as to be distinctly audible to the patient when in the horizontal attitude, and reclining on the left side. Again, accompanying the feeling of tumultuous commotion in the chest, there is often an ill-defined sensation of a roaring or rushing noise ascending towards the head, and following the course of the great vessels. This phenomenon is not observable in this case, and although we do not notice here the bellows murmur, which is said so frequently to accompany the first cardiac sound, with a little care it is possible to distinguish a faint humming sound, (*bruit de diable*,) over the great vessels of the neck. This, as you are aware, is considered to be indicative of an impoverished condition of the blood; and we see here further proof of general vascular atony, in the reluctance with which the cutaneous capillaries readmit the blood, which has been forced from them by pressure; the surface continuing, for some time, of a pale, dead white hue, instead of rapidly regaining its usual healthy tint.

The pulse, which ranges from 95 to 100 in the intervals, partakes of the same irregular and fluttering character as the impulse of the heart. During the paroxysms, it rises as high as 105 in the minute.

The paroxysms of dyspnœa continue in her case about half an hour, and recur whenever she becomes frightened or alarmed at any sudden noise, as the slamming of a door, &c., showing great irritability, and impressibility of the nervous system; and they are accompanied by constant distressing sighing, which—as I have elsewhere taught—is probably owing to the loss of balance between the amount of air inspired, and the quantity of blood thrown into the lungs, in this unusually impressible state of the cardiac centre; the result of which loss of healthy equilibrium is the insufficient aëration of the blood necessary to supply the wants of the economy; and the repeated and deep inspirations established to effect this indispensable aëration.

To obtain as clear and definite an idea as possible of the nature of the affection under which the patient is laboring, we shall endeavor to trace the cardiac disturbance to its proper origin.

Purely functional palpitation of the heart is referable to one or both of two great causes ;—defective or disturbed innervation, and derangement in the quantity or character of the circulatory fluid ; which derangement may consist in polyæmia, anæmia, or in a depraved condition of the blood ; in other words, a condition in which that fluid suffers some quantitative or qualitative change—some alteration in its sensible or chemical characters. Moreover, an obstinately deranged state of the heart, causing undue and persistent palpitation, may be the effect of previous disease. Thus, gouty and rheumatic affections often originate the morbid conditions in question ; and upon inquiry of this patient, we find, as you may have anticipated, that a few years ago she suffered from an attack of acute rheumatism.

The connection between rheumatism and cardiac disease is a matter of considerable interest, and one which has, within the present century, been much studied and investigated. You will have occasion to observe, among young persons especially, how frequently acute rheumatism is either accompanied or followed by some acute or chronic affection of the heart, and the more frequently a person is attacked with acute rheumatism the greater is the liability to organic cardiac disease.

In reviewing the history given by the patient of her malady, and the examination which we have made of the case in your presence, you will be ready to conclude that her cardiac affection is evidently functional—not organic in its character. The paroxysmal nature of the palpitation and the dyspnœa, the anæmic state, the dyspeptic phenomena which she presents, the neuralgic pain in the back, the general impressibility of the nervous system, &c., all tend to this conclusion. Our diagnosis, then, is that there is faulty innervation of the heart ; and indeed general erethism and impressibility of the whole nervous system.

The indications for treatment, under this view, are manifest. We must address our remedies to the nervous system, and endeavor to give tone and renewed vigor to the entire economy, by improving the composition of the circulating fluid, and inducing through it a new and healthy action in the tissues.

Now, the whole range of the materia medica furnishes us with nothing that will more readily effect this object than the chalybeate preparations. We might prescribe for her the Ferri pulvis of the last edition of the Pharmacopœia of the United States—*le fer réduit par l'hydrogène* of the French *pharmaciens*, which is a favorite with some physicians, but has no advantage over the Ferri subcarbonas, which I shall now order.

℞. Ferri Subcarbonat. ʒij.

Divide in chart. xii. quarum capiat unam ter in die.

The bowels are to be kept regular by an occasional dose of rhubarb and magnesia; the latter is selected as an antacid and laxative; and the former exerts, in addition to its cathartic property, a tonic influence on the alimentary tract. We shall likewise direct, that repeated and vigorous friction shall be made over the seat of pain in the back, by means of a coarse towel, or by the hand frequently dipped in wheaten flour to prevent abrasion of the surface; and shall request her to present herself at the clinic one week from this time.

Case II. Jas. McC——, æt. 41, a weaver by trade, suffers from palpitation of the heart, and severe cough, accompanied by profuse expectoration. He has for a long time been sensible of increasing ill-health, which from the detail of his symptoms is undoubtedly connected, either as cause or effect, with the cardiac derangement of which he particularly complains.

The present increase in the severity of his symptoms appears to be mainly attributable to a bronchial complication; for, in addition to his chronic malady, he is just now laboring under the effects of an attack of bronchitis; and we have purposely introduced the case to your consideration, as exemplifying not only several interesting facts connected with heart disease, but the increased difficulty encountered in arriving at a correct and satisfactory diagnosis in cases where the chronic ailment, oftentimes the original cause of the deterioration of health, is to a certain degree masked and obscured by some intercurrent affection.

Now, the most prominent of the symptoms presented by the patient, and most urgently demanding our attention and investigation, are the distressing and protracted dyspnœa—a symptom perceptible to you all, and the spitting of blood—hæmoptysis, phenomena very alarming to the patient, and playing a significant part in the formation of our diagnosis.

The patient tells you that blood is mingled with his sputa—that he spits blood. It behoves us, then, to inquire what may be the cause of this, and what relation it bears to his present or past ill-health; and furthermore, whether or not this relation, if any such exist, be ominous of evil.

Inspection of the countenance and hands reveals, by the pallid hue of the skin, a slightly anæmic condition; and coupling this circumstance with the fact, that hemorrhages are very apt to occur in consequence of diminution in the quantity, or alteration in the quality, or consistency of the blood, we might be led to attribute to some such change in the circulating fluid the passive exhalation of blood from the pulmonary mucous membrane in this case. Pulmonary hemorrhage, however, is too frequently secondary, and ascribable in the great majority of such cases to organic disease, either of the heart or lungs. A common consequence of such organic disease is hyperæmia of the pulmonary vessels, the effect of a purely mechanical agency; and this condition is often the immediate precursor of those symptomatic or passive hemorrhages as they are called. It is the direct cause of the hæmoptysis, and is itself the effect of pulmonic or cardiac lesion. Thus, the pulmonary tissue may be filled with tubercles, which so impede and obstruct the pulmonary vessels, that mechanical hyperæmia is the result, followed by hemorrhage. So, too, disease of the heart may be productive of the same effect, by obstructing the free return of the blood from the lungs. In both cases the pulmonary capillaries become turgid, and transudation or exhalation of blood very readily takes place. In such cases, it rarely, perhaps never, happens, that the parietes of the vessels do not have their nutrition and cohesion so impaired as to render the escape of blood more easy.

On ausculting the patient, we find, that the inspiratory murmur is not continuous, as in health, but is interrupted, as by jerks or starts. This is very appropriately called the "interrupted or jerking respiration,"—*respiration saccadée* of the French writers, and is generally regarded as an unfavorable symptom, indicating as it often does, incipient tuberculous deposition. It ought usually to be looked upon as such; but as it would seem to be an accompaniment of pleurisy, pleurodynia, and spasmodic asthma, in their incipiency, you will readily see, that its diagnostic value depends much upon the concomitant phenomena.

The patient complains of continued and distressing dyspnœa, the cause of which requires investigation; as difficulty of breathing is a phenomenon met with under a variety of circumstances, and may be caused by many different morbid conditions.

Physiologically regarded, dyspnœa is experienced whenever the normal relation between the amount of air inspired, and the quantity of blood circulating through the lungs, is in any manner disturbed. An effort,—oftentimes an exceedingly severe and distressing one,—is required to preserve this equilibrium. Whatever, therefore, prevents the proper aëration of the blood is a cause of dyspnœa. A little further reflection will show you, that diseases of the heart and lungs must be frequently attended with this uncomfortable symptom, in consequence of the obstacle which, in a greater or less degree, they offer to this indispensable process.

Undue activity of the circulation, faulty innervation of the respiratory muscles, pleurisy, emphysema, hepatization and tuberculization of the lungs, or any condition causing obliteration of the air cells, or excluding the air from any portion of the lungs, as in pneumonia, pulmonary hemorrhage, &c., where the bronchi and air vesicles sometimes become choked with blood and mucus; disease of the cardiac valves impeding the passage of blood into or obstructing its egress from the lungs, effusion into the pericardium, &c., for the reasons stated, are all more or less accompanied with dyspnœa.

Though the original difficulty of breathing was probably connected with the cardiac disturbance, the present increased dyspnœa, we are inclined to think, is mainly traceable to a pulmonary cause. Percussion aids us here; for we find, on examination, a very slight, but sufficiently perceptible, dulness under the left clavicle. This is unfavorable, inasmuch as when taken in connection with the jerking character of the respiration, the hæmoptysis, the dyspnœa, &c., it augurs incipient tuberculosis.

The rest of the chest is clear.

Auscultation reveals great obscurity in the sounds of the heart; it is indeed almost impossible to discriminate them; and this, in part, is owing to the mucous rhonchi, which may be heard even before the ear reaches the chest of the patient; and by listening attentively, they are found to be intermixed with sonorous and sibilant rhonchi. The air enters the lungs freely and readily on the right side: on the left, less so; and we have the slight cooing and violoncello varieties of the sonorous rhonchi.

Of all the affections with which heart-disease is associated, pulmonary complications are the most frequent, and generally require the most energetic treatment. Bronchitis, pneumonia, and pleurisy are often co-existent diseases; sometimes superadded by the action of extraneous causes; and very often, it must be admitted, as effects of the cardiac derangement. So blended and intermingled, as in the present case, are the symptoms, at times, that it is extremely difficult and occasionally impossible to determine the exact nature of the two morbid conditions, and their relations to each other, or to ascertain to which is due the priority of occurrence.

Pulmonary hyperæmia resulting from cardiac disease may terminate, as is often the case, by the exhalation of blood from the mucous lining of the air passages; or it may run on so as to produce an inflammatory condition of the bronchial lining, or of the pulmonary tissue, and thus complicate the primary cardiac disease.

In consideration of the tuberculous tendency evinced by this patient, we shall put him on the use of the oleum morrhuæ, in the dose of a tablespoonful three times a day; and in addition thereto, with a view to its mixed eutrophic and tonic effects, direct him to take at the same time, ten drops of the liquor ferri iodidi, morning, noon and night, in a wineglassful of simple syrup.

In chronic diseases of the heart but little benefit may be expected from counter-irritation; but in a case like this, where pulmonic complication exists, a resort to some revellent agent, is indicated. We shall direct him to rub upon his breast, night and morning, the oleum tiglii, mixed with an equal portion of the oleum olivæ, until the peculiar eruption is produced; when the application must be discontinued, until the eruption has passed away. It may then be resumed. He will thus have all the benefit which interrupted counter-irritation is capable of effecting. As his cough is troublesome, and by its severity tends to keep up the hæ-

moptyis, he may take in the course of the day, in divided doses, a teaspoonful of the liquor morphiæ sulphatis, added to the ordinary domestic cough mixture—Hausmittel—of equal parts of vinegar, butter and molasses stewed together, which we are in the habit of prescribing in this clinic.

(To be concluded in next Number.)

